# Combinatorial Cytotoxic Effects of Gelam Honey and 5-Fluorouracil against Human Adenocarcinoma Colon Cancer HT-29 Cells In Vitro

**DOI:** 10.1155/2019/3059687

**Published:** 2019-02-21

**Authors:** Syed Ahmad Tajudin T-Johari, Fatimah Hashim, Wan Iryani Ismail, Abdul Manaf Ali

**Affiliations:** ^1^School of Fundamental Science, Universiti Malaysia Terengganu, 21030 Kuala Terengganu, Malaysia; ^2^Faculty of Bioresources and Food Industry, Universiti Sultan Zainal Abidin, 22200 Besut, Terengganu Darul Iman, Malaysia; ^3^Institute of Marine Biotechnology, Universiti Malaysia Terengganu, 21030 Kuala Terengganu, Terengganu Darul Iman, Malaysia; ^4^Research Institute of Agriculture Production and Food Innovation, Universiti Sultan Zainal Abidin, 22200 Besut, Terengganu Darul Iman, Malaysia; ^5^Natural Medicine Research Centre, Universiti Islam Malaysia, 63000, Cyberjaya, Selangor Darul Ehsan, Malaysia

## Abstract

Combination of natural products with chemodrugs is becoming a trend in discovering new therapeutics approach for enhancing the cancer treatment process. In the present study, we aimed to investigate the cytotoxic and apoptosis induction of Gelam honey (GH) combined with or without 5-Fluorouracil (5-FU) on HT-29 cells. The cell viability was determined by 3-(4,5-dimethylthiazol-2-yl)-2,5-diphenyltetrazolium bromide assay to assess cytotoxicity. Morphological changes and apoptosis were determined by the inverted microscope, Annexin V-FITC, and DNA fragmentation via flow cytometric analysis, respectively. Our results demonstrate that combined treatment revealed a remarkable and concentration-dependent cytotoxic effect on HT-29 cells in comparison with GH and 5-FU alone. Flow cytometry analysis showed that early apoptosis event was more pronounced in combined treatment. In addition, compared to 5-FU alone, apoptosis of HT-29 cells treated with combinations of GH and 5-FU demonstrated increasing percentages of fragmented DNA. Our results suggest that GH has a synergistic cytotoxic effect with 5-FU in HT-29 cell lines* in vitro*. Although the actions of the molecular mechanisms are not yet clear, the results reveal that the combination of GH and 5-FU could have the potential as a therapeutic agent.

## 1. Introduction

Since antiquity, honey has been consumed as a daily nutritional supplement. Its major constituent is carbohydrates such as glucose, fructose, and sucrose. Honey bees collect pollen from flowers and later convert them into honey via regurgitations and evaporations. There are several varieties of honey in Malaysia, such as Honey, Tualang Honey, and Pineapple Honey. All of these are differentiated based on their dominant quantity of pollen, which can be identified by pollen analysis as the pollen is species-specific [1]. Honey is scientifically proven to have several medicinal properties such as antimicrobial [2, 3], antioxidant [4], anti-inflammatory [5], antitumour [6], and wound healing abilities [7]. Phenolic compounds inside honey, such as gallic acid, chlorogenic acid, caffeic acid, p-coumaric acid, ferulic acid, ellagic acid, quercetin, hesperetin, and chrysin, are the major contributors to the anti-inflammatory and antitumour effects of honey [6, 8].

During the initial treatment of colorectal cancer, a chemotherapeutic drug known as 5-Fluorouracil (5-FU) is usually used. Its main mechanism involves the disruption of the normal functions of DNA and RNA via the misincorporation of fluoronucleotide into sequence, apart from inhibiting the function of thymidylate synthase [9]. However, 5-FU has been reported to be of low availability within the cells due to its degradation in the liver by the enzyme dipyrimidine dehydrogenase (DPD). Thus, a large dose is required during treatment [10]. Higher doses of this drug can cause severe side effects to the patients in addition to being very toxic to the human body. Past studies have found that combining the drug with natural substances such as honey can enhance its effect on cancerous cells and minimise its toxicity [11]. The usage of GH in combination with 5-FU has been shown to significantly reduce the growth of HCT-116 cells, unlike treatment with 5-FU alone [12]. In addition, GH plus ginger extracts has exhibited synergistic effects on HT-29 cells in terms of the upregulation of caspase-9 expression [13].

In this study, Gelam honey, 5-Fluorouracil, and their combination were used to determine the cytotoxic and apoptotic effects on HT-29 cells. Observations were made in terms of changes in the membrane integrity, fragmentation of DNA, and early events of apoptosis. These are useful for the creation of new strategies for the future treatment of colorectal cancer.

## 2. Materials and Methods

### 2.1. Honey Sample

The Gelam honey used in this study was obtained from Gelam Forest, Besut, Terengganu, Malaysia. Stock solution of honey was prepared by mixing the honey with RPMI-1640 medium and filter-sterilising using a 0.22-*μ*m syringe filter. The Gelam honey used in this study has been tested by an accredited laboratory and confirmed to be pure honey.

### 2.2. Cell Line

Human colorectal adenocarcinoma HT-29 cells were obtained from American Tissue Culture Collection (Manassas, VA, USA). The cells were grown in RPMI-1640 medium (Sigma, St. Louis, USA), supplemented with 10% foetal bovine serum (GIBCO, USA) and antibiotics (i.e., 100.0 units/mL penicillin and 100.0 *μ*g/mL streptomycin) (PAA, Austria). They were maintained in an incubator at 37°C with 5% CO_2_ and a humidified environment. The HT-29 cells were subcultured every 2 to 3 days in a semiconfluent condition in which they were treated with a trypsin-like enzyme and phenol red (GIBCO, USA) for 5 minutes. The cells were then resuspended in the medium with serum before being transferred into 2 or 3 new flasks. Samples with cell viability of 95% and above were selected for use throughout this study.

### 2.3. MTT Cytotoxicity Assay

The MTT assay was carried out in a 96-well plate, as described by Ali et al. [14]. A 100.0 *μ*L of complete growth medium was placed into 96 flat-bottom microtiter plate (Nunclon, USA). This was followed by the addition of 100.0 *μ*L of HT-29 cells at concentrations of 1-2 × 10^5^ cells/mL that have been seeded for 24 h prior to usage. Honey samples (400 mg/ml) in RPMI-1640 medium were aliquoted into the wells in triplicate and serially diluted. Untreated cells were used as a control. The plate was incubated for 72 h in a CO_2_ incubator at 37°C. After incubation, 20.0 *μ*L of MTT solution (5.0 mg/mL) was added to each well and further incubated for 4 h. The culture medium was then removed from the wells and 100.0 *μ*L of dimethylsulphoxide (DMSO) added to each well to solubilise the resulting formazan [15]. The optical densities (OD) of the wells were analysed at 570 nm using a plate reader (BIOTEK, USA), with a reference at 630 nm. A dose-response curve of cell viability versus sample concentration was subsequently plotted.

### 2.4. Acridine Orange and Propidium Iodide Staining (AO/PI) Analysis

The HT-29 cells were treated with GH, 5-FU, and a combination of both for 24, 48, and 72 h. After being incubated for 24 h, the cells were harvested into centrifuge tubes and pelleted down at 300 × g for 10 min. The cell pellets were washed with PBS by centrifuging as mentioned above. The pellets were then suspended in 50.0 *μ*L of acridine orange (10.0 *μ*g/mL) and 50.0 *μ*L of propidium iodide (10.0 *μ*g/mL) for 5 min. A volume of 10.0 *μ*L of stained cells was pipetted onto a glass slide and covered with a cover slip. The viable, apoptotic, and necrotic cells were scored in populations of more than 100 cells using an inverted fluorescence microscope (Nikon TE2000-U, Nikon, Japan), as described by Ali et al. [16]

### 2.5. Phosphatidylserine Externalisation Analysis by Flow Cytometry

An Apoptosis Detection Kit (BD Annexin V-FITC) was used for flow cytometry analysis. The kit contained Annexin V conjugated with fluorochrome FITC, propidium iodide, and a binding buffer. HT-29 cells (2 × 10^5^ cells/mL) were treated with GH, 5-FU, and a combination of both in 6 wells for 3, 6, and 12 h. Following the completion of treatment, the cells were harvested into 5-mL tubes and centrifuged at 300 × g in a swing rotor for 10 minutes. The cell pellets were washed twice with PBS, after which 100 *μ*l of binding buffer was added to the tubes. A volume of 5 *μ*l of Annexin V FITC and 5 *μ*l of PI were added to the tubes as a staining solution. The mixture was then incubated in the dark for 15 minutes. This was followed by addition of 400 *μ*l of binding buffer to each tube. The tubes were gently vortexed prior to analysis using a CytoFLEX flow cytometer (Beckman Coulter, USA). Approximately 10,000 events were sorted accordingly into viable, early apoptotic, late apoptotic, and necrotic cells stages [17, 18].

### 2.6. DNA Fragmentation Analysis by Terminal Deoxynucleotidyl Transferase dUTP Nick-End Labelling (TUNEL)

TUNEL assay was carried out using APODIRECT™ Kit in accordance with the manufacturer's protocol (Becton Dickinson, USA). Cells at the concentration 1 × 10^6^ cells/mL were fixed in 1% (w/v) paraformaldehyde with PBS (pH 7.4) for 60 minutes on ice. The cells were then pelleted by centrifugation at 300 × g for 5 minutes before being washed twice with 5 mL of PBS. The cells were then resuspended in 70% (v/v) ice-cold ethanol and stored for 1 week at -20°C. After incubation, the ethanol was removed and the cells were washed twice using a washing buffer. The cells were then subjected to end-labelling by incubation in 50 *μ*l of a DNA Labelling Solution (containing TdT Enzyme and FITC dUTP dissolved in the reaction buffer) for 60 minutes at 37°C. Following incubation, the cells were washed twice in 1 mL of rinse buffer prior to staining with 0.5 mL of PI/RNase Staining Buffer. The staining process was carried out for 30 minutes in a dark environment. After that, the cells were then analysed in CytoFLEX (Beckman Coulter, USA) using the CytExpert software.

### 2.7. Statistical Analysis

The numerical parameters were expressed as means ± standard error means (S.E.M.). All experiments were performed in triplicate and analysed using one-way analysis of variance (ANOVA) followed by Tukey's post hoc test. Values with confidence levels of p ≤0.05 were considered to be statistically significant.

## 3. Results

### 3.1. Cytotoxicity of Gelam Honey, 5-Fluorouracil, and Their Combination in HT-29 Cells and Normal Colon Cells

**Figure 1:**
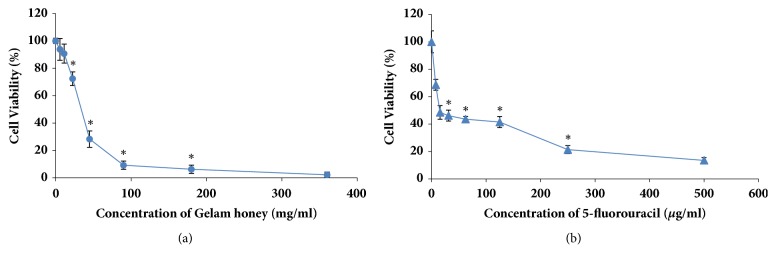
Cytotoxicity of GH and 5-FU against HT-29 cells after 72 h of treatment. The CD_50_ of GH was 36.2 mg/ml (a) and the CD_50_ of 5-FU was 15.5 *μ*g/ml (b). Each point represents the mean of the results of three independent experiments. Error bars represent means ± S.E.M. of the three independent experiments.*∗*Statistical significance (P<0.05) between control cells and treatment groups.

**Figure 2:**
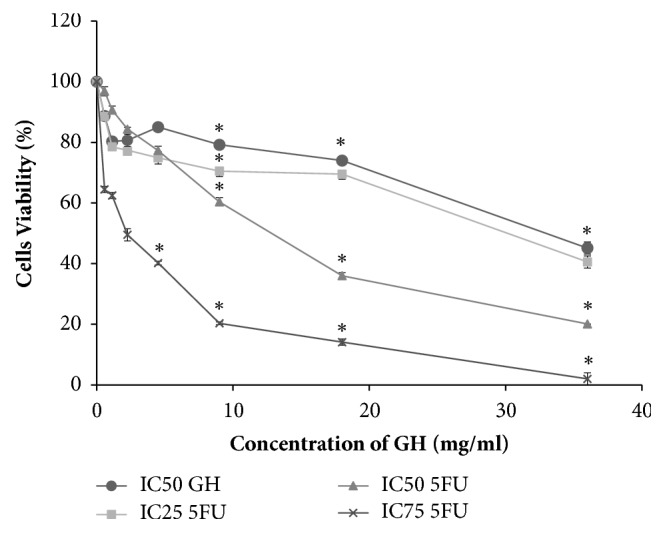
Effects of GH at CD_50_ concentration on the viability of HT-29 cells after incubation in different concentrations of 5-FU for 72 h. Each point represents the mean of the results of three independent experiments. Error bars represent means ± S.E.M. of the three independent experiments. *∗*Statistical significance (P<0.05) between control cells and treatment groups.

**Figure 3:**
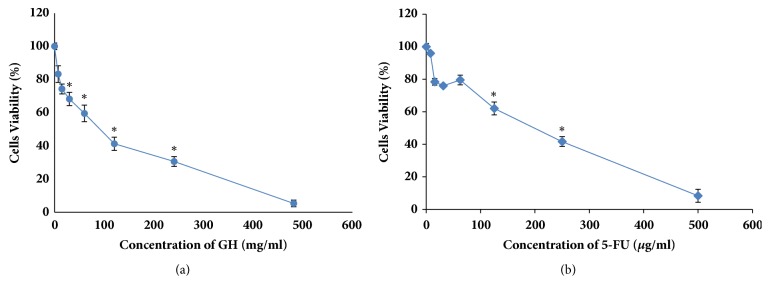
Cytotoxicity of GH and 5-FU against normal colon cells (ATCC® CRL-1459) after 72 h of treatment. The CD_50_ of GH was 89.8 mg/ml (a) and the CD_50_ of 5-FU was 203.1 *μ*g/ml (b). Each point represents the mean of the results of three independent experiments. Error bars represent means ± S.E.M. of the three independent experiments. *∗*Statistical significance (P<0.05) between control cells and treatment groups.

Gelam honey was examined for its cytotoxicity towards HT-29 cells using a MTT assay. The optical density of the resulting formazan blue was determined, which reflected the normal function of mitochondrial dehydrogenase in viable cells [15]. The degrees of toxicities of GH, 5-FU, and their combination after 72 h treatment (with respect to the untreated cells) were determined according to their respective concentrations that reduced the cell populations to 50% (CD_50_). Gelam honey reduced the number of viable HT-29 cells at a CD_50_ of 36.2 mg/ml. Meanwhile, the CD_50_ of 5-FU was 15.5 *μ*g/ml (Figure 1). From the results, the CD_25_ and CD_75_ of 5-FU were 6.25 *μ*g/ml and 227.4 *μ*g/ml, respectively. The combination of CD_50_ concentration of GH with three different concentrations (CD_25_, CD_50_, and CD_75_) of 5-FU reduced the CD_50_ of GH to 30.4 mg/ml, 13.2 mg/ml, and 2.4 mg/ml, respectively (Figure 2). The cytotoxicities of GH and 5-FU were also tested on a normal colon cell line, CCD-18Co. The CD_50_ value of GH in CCD-18Co cells was 89.8 mg/ml. Meanwhile the CD_50_ of 5-FU in CCD-18Co cells was 203.1 *μ*g/ml (Figure 3). Therefore, the combination of 36.2 mg/ml GH and 15.5 *μ*g/ml 5-FU was chosen as both values were below the CD_50_ values of GH and 5-FU in normal colon cells. The combination index was calculated to ascertain the synergistic activity of the combination of GH and 5-FU [19]. The combination index (CI) analysis value obtained was less than 1, indicating that there was a synergistic effect between GH and 5-FU. The CD_50_ values of GH, 5-FU, and their combination (all at CD_50_ concentrations) were used in every subsequent assay of this study.

### 3.2. Mode of Cell Death by Dual-Staining with Acridine Orange and Propidium Iodide

**Figure 4:**
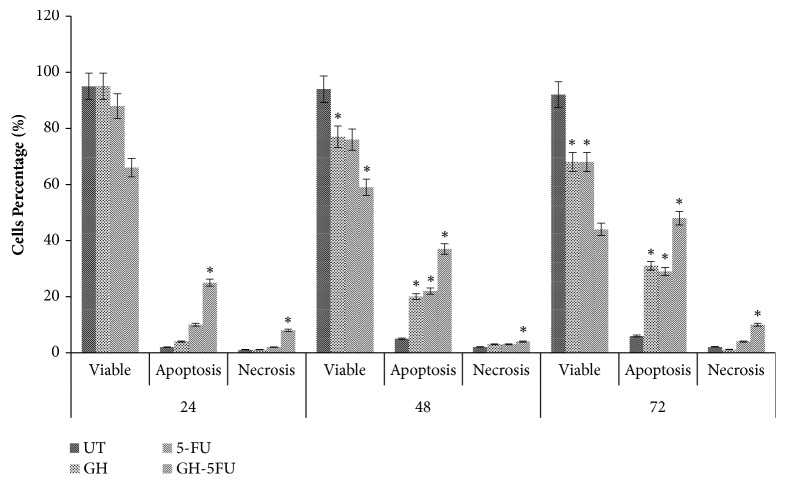
Mode of cell death of untreated HT-29 cells and those which have been treated with GH, 5-FU, and both. Error bars represent means ± S.E.M. of the three independent experiments. *∗*Statistical significance (p<0.05) between control cells and treatment groups.

Morphological assessments of the cells (using acridine orange and propidium iodide) were conducted to determine the modes of death of the treated HT-29 cells at CD_50_ doses. As shown in Figure 4, the number of apoptotic cells increased significantly after treatment with GH from 20.67% at 24 h to 34.67% at 48 h and 41.03% at 72 h. Similarly, in the positive control (5-FU-treated cells), the corresponding values were 7.67% at 24 h, 22.02% at 48h, and 23.01% at 72 h. The highest percentages of apoptotic cells were recorded for the combined treatment of GH + 5-FU, with values of 25.33% at 24 h, 33.05% at 48 h, and 46.67% at 72 h. A low percentage of necrotic cells was detected in the treatment groups, which did not differ significantly from the untreated group.

### 3.3. Phosphatidylserine Externalisation Analysis in Terms of Early Apoptotic Events

Phosphatidylserine externalisation is an early event during apoptosis, which can be evaluated by using Annexin V and PI staining. In this study, it was found that this early event of apoptosis was detected in all groups within 3 h of treatment at CD_50_ dose (Figures 6 and 7). For the GH-treated specimens, the percentages of early apoptotic events were 4.66% at 3 h, 6.20% at 6 h, and 12.31% at 12 h. The percentages of late events measured were 4.5% at 3 h, 3.85% at 6 h, and 10.16% at 12 h. No events of necrosis were detected in the GH-treated specimens. Similar measurements were conducted for the 5-FU-treated specimens. The percentages of early apoptotic events in the 5-FU-treated specimens increased over time (6.66% at 3 h, 8.11% at 6 h, and 16.56% at 12 h). However, there were lower percentages of late apoptotic events in the 5-FU-treated specimens as compared to the GH-treated groups (4.41% at 3 h, 3.05% at 6 h, and 6.79% at 12 h). Higher percentages of apoptotic cells were obtained in the combined (GH + 5-FU) treatment group as compared to the either GH or 5-FU alone. In the combined treatment, the percentages of early apoptotic events were 7.51% at 3 h, 9.37% at 6 h, and 18.64% at 12h.

### 3.4. DNA Fragmentation Analysis: Hallmark Apoptosis Detection

DNA fragmentation is one of the hallmark events of apoptosis. A quantitative evaluation of DNA fragmentation in the cells was performed using TUNEL assay. After a 24 h treatment period, the DNA inside the HT-29 cells started to undergo cleavage in all treatment groups (with the percentages of such cells being 4.44% in GH treatment, 4.21% in 5-FU treatment, and 71.67% in the combined GH + 5-FU treatment).  Figure 8 shows a significant increase in the percentage of TUNEL-positive cells in the combined GH + 5-FU treatment group (92.49% after 48 h and 94.90% after 72 h). It was noted that all treatments at CD_50_ doses increased the percentages of TUNEL-positive cells with time. In contrast, the control groups recorded a very low incidence of DNA cleavage (less than 0.3%) throughout the treatment period. This showed that DNA cleavage was not a normal occurrence in healthy HT-29 cells, hence suggesting that the cause of the same was the cytotoxicities of GH and 5-FU.

## 4. Discussion

Over the past few years, cancer chemoprevention and therapy using traditional medicines have garnered a great deal of research attention globally. In this study, the cytotoxic and apoptosis induction of Gelam honey (GH) combined with or without 5-Fluorouracil (5-FU) on HT-29 cell were investigated. The cytotoxic result revealed that GH exhibited the ability to reduce the viability of HT-29 cells, and in combined treatment the cytotoxic dose reduced compared with GH and 5-FU alone in time and dose dependent manner. The results also indicated synergistic effect of GH and chemoagent 5-FU in altering growth of HT-29 cells, which was quantitatively analysed according to Chou-Talalay method [19]. Previous study showed that GH has high levels of total phenolic compound which correlated significantly to its bioactivity [20]. It is also found that phenolic compounds in honey contribute directly to the reduction of the colon cancer cells viability [21]. Similar effects could not be seen in normal colon cells, suggesting that GH activity is cell dependent and therefore has the potential to be used as alternative for colon cancer therapy in combinations with 5-FU. 5-FU is a well-known drug and widely used in colon chemotherapy. Numerous strategies have been established to improve the activity of 5-FU, one of which is the use of combination therapy [22–24]. In common clinical practice, the combination of 5-FU with leucorin, irinotecan, or oxaliplatin has shown improvements of this drug activity [25] However, side effects associated with 5-FU might hamper the effort to treat cancer patients [26]. With the aim of highlighting the hypothesis that GH could possibly enhance the 5-FU activity, the combinations of GH and 5-FU initiated by Hakim et al. revealed that GH can be a potential candidate to work synergistically with 5-FU [12]. However, the discovery of its potential value was not sufficient and more data need to be acquired to reveal its benefits. To the best of our knowledge, no experimental evidence to date has shown the association of Gelam honey and 5-FU in inducing apoptosis in HT-29 cells, respectively. Nevertheless, researches on the bioactivity of Gelam honey alone were intensively being done. Gelam honey is a well-known prominent substance, inexpensive, relatively nontoxic, and easily available in marketplace. Furthermore, honey as regards its origin has been used since the ancient times. Gelam honey has been considered as a potential cancer therapy that has the ability to reduce the viability various cancerous cells line such as MCF-7, HCT-116, HepG2, A549, and HT-29 [12, 27–30].

**Figure 5:**
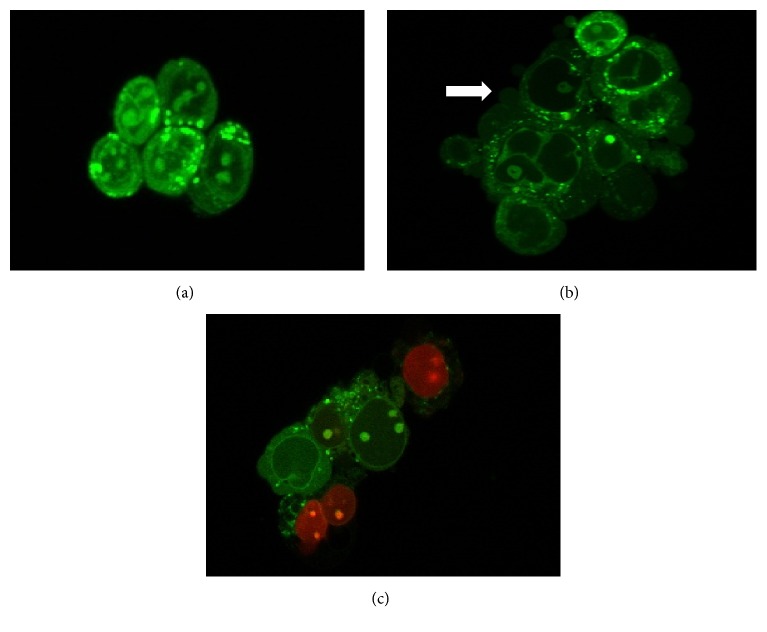
Fluorescent photomicrographic evidence of HT-29 cells 24 h after treatment. Morphological changes following exposure to treatment are typical of apoptosis; A: viable cell, B: apoptotic cell, C: necrotic cell. The arrow (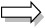) shows membrane blebbing—one of the characteristics of apoptosis. This was viewed using a laser confocal inverted microscope at magnification of 630X.

**Figure 6:**
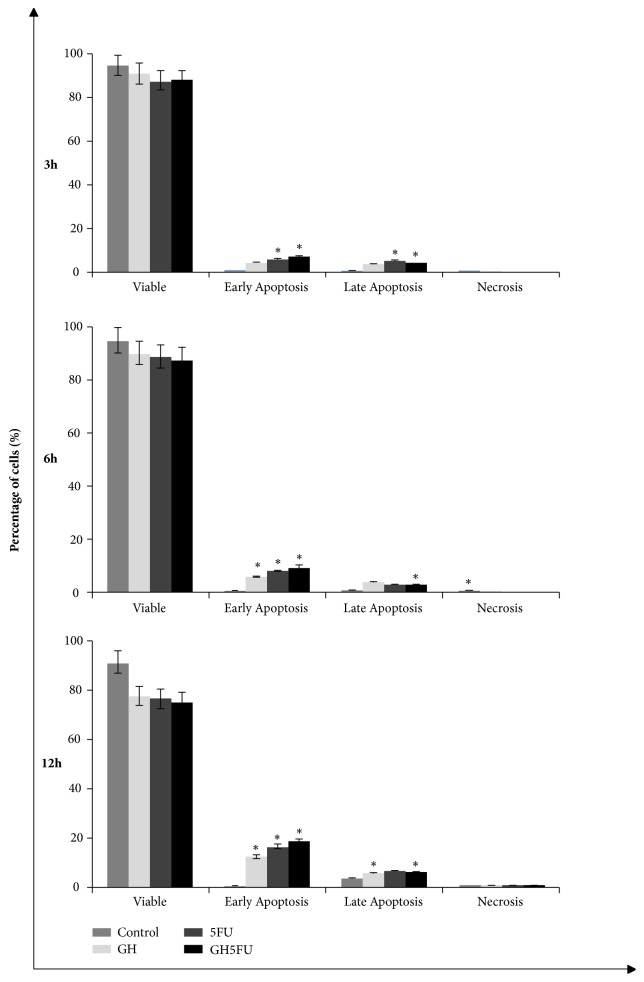
Analysis of phosphatidylserine externalisation in HT-29 cells via Annexin V and PI staining using a flow cytometer. Results are presented as means ± S.E.M. of three independent experiments. *∗*Statistical significance (P<0.05) between control cells and treatment groups.

**Figure 7:**
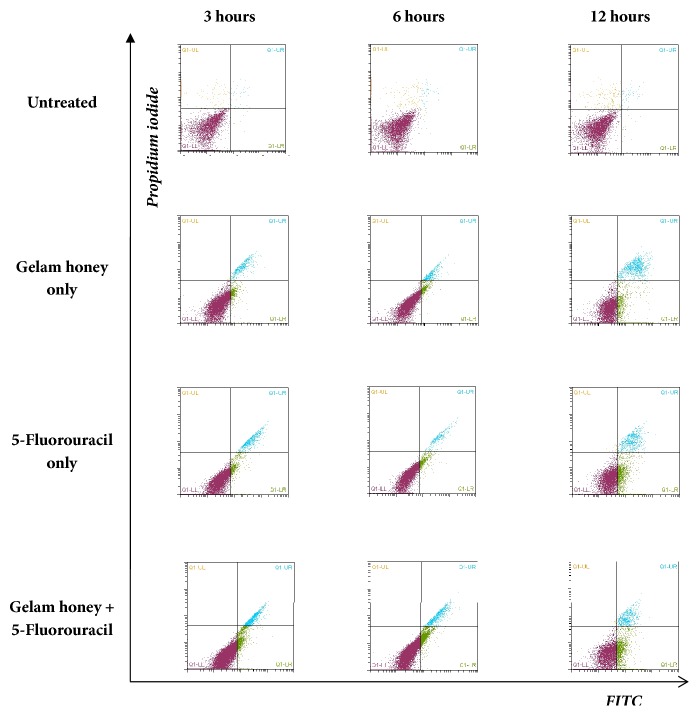
Flow cytometry analysis of untreated and CD_50_-treated HT-29 cells via staining with Annexin V FITC/propidium iodide (PI). Viable cells are in the lower left quadrant, early apoptotic cells are in the lower right quadrant, late apoptotic cells are in the upper right quadrant, and nonviable necrotic cells are in the upper left quadrant. Each dot in the plot represents 10,000 cells in a single replicate.

**Figure 8:**
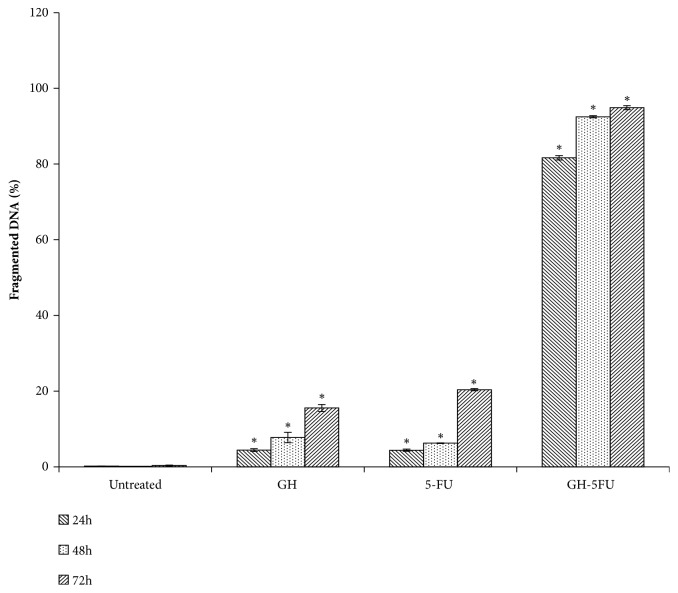
DNA fragmentation of HT-29 cells after treatment with GH, 5-FU, and both. Results are expressed as percentages of cells stained by terminal dUTP nick-end labelling (TUNEL positive). Error bars represent means ± S.E.M. of the three independent experiments. *∗*Statistical significance (P<0.05) between control cells and treatment groups.

Many studies in vitro and in vivo showed that GH alone has significant antiproliferative and apoptotic effects observed in cancer cells treated by GH [13, 27, 28, 30, 31]. In cancer cells, the induction of apoptosis is known to be an efficient strategy for cancer therapy. Apoptosis is a programmed cell death that becomes focused in the field of cell death which involved either intrinsic (mitochondria dependent) or extrinsic (death receptor dependent) pathway [32–34]. During early event of apoptosis, translocation of phosphatidylserine (PS) from inner surface of the cell membrane to outer cell membrane occurred [35, 36]. We demonstrated that the phosphatidylserine (PS) was translocated as early as 3 hours after exposing the HT-29 cells to the treatment. Interestingly, the combined treatment showed that the highest percentage of PS was translocated in cells. Even though the translocation of PS has been reported in reversible process [37], our morphology examination showed that the cells treated in the combination treatment experienced blebbing characteristics with an increasing number of cells compared to a single treatment (Figure 5). These confirm that HT-29 cells undergo apoptosis and are not reversible. In addition, our result in DNA fragmented analysis showed that the combination of GH and 5-FU increased the fragmented DNA in HT-29 cells. DNA fragmentation is one of the late stage processes occurring in apoptosis pathway [38, 39]. Collectively, these findings further support the idea that cytotoxic effect of GH and 5-FU and combinations of both induced apoptosis in HT-29 cells in a time-dependent manner.

## 5. Conclusion

In conclusion, the combination of GH and 5-FU had a synergistic outcome with respect to the cytotoxic and apoptotic effects on human colorectal cancer HT-29 cells. The synergistic interaction of 5-FU and GH on HT-29 cells warrants further investigation of the underlying molecular mechanism on HT-29 cells where focus should be on the molecular interaction to find out specifically in which pathway GH enhances the efficacy of 5-FU.

## Data Availability

The data used to support the findings of this study are included within the article.
