# Corrigendum to “Combinatorial Cytotoxic Effects of Gelam Honey and 5-Fluorouracil against Human Adenocarcinoma Colon Cancer HT-29 Cells In Vitro”

**DOI:** 10.1155/2019/9050626

**Published:** 2019-07-18

**Authors:** Syed Ahmad Tajudin T-Johari, Fatimah Hashim, Wan Iryani W. Ismail, Abdul Manaf Ali

**Affiliations:** ^1^School of Fundamental Science, Universiti Malaysia Terengganu, 21030 Kuala Terengganu, Malaysia; ^2^Faculty of Bioresources and Food Industry, Universiti Sultan Zainal Abidin, 22200 Besut, Terengganu Darul Iman, Malaysia; ^3^Institute of Marine Biotechnology, Universiti Malaysia Terengganu, 21030 Kuala Terengganu, Terengganu Darul Iman, Malaysia; ^4^Research Institute of Agriculture Production and Food Innovation, Universiti Sultan Zainal Abidin, 22200 Besut, Terengganu Darul Iman, Malaysia; ^5^Natural Medicine Research Centre, Universiti Islam Malaysia, 63000, Cyberjaya, Selangor Darul Ehsan, Malaysia

In the article titled “Combinatorial Cytotoxic Effects of Gelam Honey and 5-Fluorouracil against Human Adenocarcinoma Colon Cancer HT-29 Cells In Vitro” [1], the name of the third author was given incorrectly as Wan Iryani Ismail. The author's name should have been written as Wan Iryani W. Ismail. The revised authors' list is shown above.

## References

[1] T-Johari S. A. T., Hashim F., Ismail W. I., Ali A. M. (2019). Combinatorial cytotoxic effects of gelam honey and 5-fluorouracil against human adenocarcinoma colon cancer HT-29 cells in vitro. *International Journal of Cell Biology*.

